# Oesophageal Stem Cells and Cancer

**DOI:** 10.1007/978-3-319-69194-7_10

**Published:** 2017-12-05

**Authors:** Maria P. Alcolea

**Affiliations:** 7 Wellcome Trust-Medical Research Council Cambridge Stem Cell Institute, Tennis Court Road, CB2 1QR Cambridge, UK; 8 Department of Oncology, University of Cambridge, Hutchison/MRC Research Centre, Hills Road, CB2 0XZ Cambridge, UK

**Keywords:** Oesophageal cancer, Oesophageal stem cells, Oesophageal models, Lineage tracing, Early tumorigenesis

## Abstract

Oesophageal cancer remains one of the least explored malignancies. However, in recent years its increasing incidence and poor prognosis have stimulated interest from the cancer community to understand the pathways to the initiation and progression of the disease.

Critical understanding of the molecular processes controlling changes in stem cell fate and the cross-talk with their adjacent stromal neighbours will provide essential knowledge on the mechanisms that go awry in oesophageal carcinogenesis. Advances in lineage tracing techniques have represented a powerful tool to start understanding changes in oesophageal cell behaviour in response to mutations and mutagens that favour tumour development.

Environmental cues constitute an important factor in the aetiology of oesophageal cancer. The oesophageal epithelium is a tissue exposed to harsh conditions that not only damage the DNA of epithelial cells but also result in an active stromal reaction, promoting tumour progression. Ultimately, cancer represents a complex interplay between malignant cells and their microenvironment. Indeed, increasing evidence suggests that the accumulation of somatic mutations is not the sole cause of cancer. Instead, non-cell autonomous components, coming from the stroma, can significantly contribute from the earliest stages of tumour formation.

The realisation that stromal cells play an important role in cancer has transformed this cellular compartment into an attractive and emerging field of research. It is becoming increasingly clear that the tumour microenvironment provides unique opportunities to identify early diagnostic and prognostic markers, as well as potential therapeutic strategies that may synergise with those targeting tumour cells.

This chapter compiles recent observations on oesophageal epithelial stem cell biology, and how environmental and micro-environmental changes may lead to oesophageal disease and cancer.

## Outline

Oesophageal tissue maintenance, self-renewal and regenerative potential remains a largely unexplored field in epithelial stem cell biology. However, the increasing incidence and poor prognosis of oesophageal cancer have stimulated interest from the cancer and stem cell community to understand the cellular and molecular mechanisms underlying oesophageal stem cell biology, and howAlcolea, M.P. dysregulation of tissue homeostasis can lead to epithelial diseases such as cancer.

Evidence indicates that environmental cues represent an important factor in the aetiology of oesophageal carcinogenesis. The Oesophageal epithelium is a tissue exposed to harsh environmental conditions; alcohol and tobacco consumption as well as gastric refluxate represent only a portion of the aggressions that the oesophagus has to endure. This certainly dictates the way this tissue is maintained and functions, and makes it susceptible to the accumulation of genetic mutations and the development of cancer.

In this chapter, I will revise recent observations in oesophageal epithelial stem cell biology, and how environmental changes may lead to oesophageal disease and cancer.

## Oesophagus

**Fig. 10.1:**
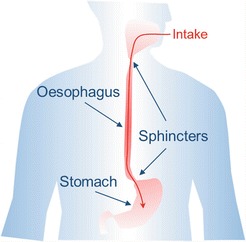
Representation of the Human oesophagus. This tissue has a simple anatomy; it represents a tube that connects our external environment with our stomach. Sphincters ensure a unidirectional transport of ingested material under normal conditions

The oesophagus is a relatively uncomplicated tube that connects our external environment with our stomach, providing means to transport food and liquids for their subsequent digestion and absorption into our bodies (Fig. 10.1). Although this organ forms part of the gastrointestinal tract, its mereAlcolea, M.P. function is to transport ingested substances unidirectionally, no food processing or absorption happens here (Goetsch 1910).

Given its Oesophageal stem cell Piping function, the architecture of this organ is relatively simple compared to other gastrointestinal organs such as the stomach and the intestine. Although histological differences exist between different animals, the oesophagus is constituted by a layer of epithelial tissue or mucosa at the outer lumen side, underlying submucosa where vascular and connective tissue can be found, and the muscularis external. This muscularis muscle layer grades from skeletal to smooth muscle towards the stomach side of the oesophagus. This muscular grading allows for voluntary swallowing to become a reflex towards the end of the oesophagus, ensuring food or drink to be delivered to the stomach for digestion. At the gastroesophageal junction the sphincter prevents reflux guarantying unidirectional transport (Goetsch 1910).

## Environment

The outer most side of the oesophagus, the mucosa or oesophageal epithelium, is in direct contact with the outsideAlcolea, M.P.. Of the gastrointestinal track, this and the epithelial mucosa of the oral cavity will be the part more directly exposed to unprocessed ingested material. This ranges from relatively high temperature products like hot tea infusions or coffee, to cold drinks, environmental pollutants, including cigarette smoke in case of smokers, alcohol consumption and chemicals such as drugs but also endless food preservatives, colouring and texturizing agents (Lin et al. 2016; Tetreault 2015; Fitzgerald 2005). All this is aggravated by the constant physical abrasion of the tissue by undigested food fragments.

The constant wear and tear to which this tissue is exposed necessitates a resistant lining to ensure functionality, endurance and, ultimately, survival. This is achieved by a squamous epithelium formed by several layers of epithelial cells with high turnover frequency that stratify towards the surface forming a multi-layered highly resilient tissue (Alcolea and Jones 2015). Studies using thymidine analogue incorporation in patients have suggested a turnover of approximately 11 days for healthy human oesophageal epithelium, double that of the intestine (Pan et al. 2013). Epithelial cells proliferate at the base of the tissue, and subsequently differentiate stratifying toward the tissue surface where they terminally differentiate and eventually shed at the outer lumen side (Barbera et al. 2015). This represents an excellent way to keep renewing cells potentially damaged by exposure to environmental factors.

However, even though the oesophageal epithelial lining is able to resists and face most day-to-day aggressions, when abused the epithelium may suffer damage and result in oesophageal disease or even cancer. One clear example of this is acid reflux (Fitzgerald 2005).

**Fig. 10.2:**
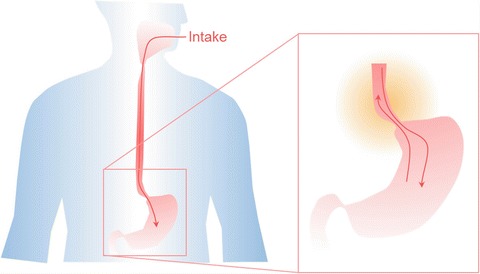
Ingested material is normally transported unidirectionally. Under certain circumstances, gastric reflux occurs exposing the oesophageal epithelium to stomach secretions that sensitize the tissue. Continued exposure may result in epithelial metaplasia, transforming this squamous tissue in intestinal-like columnar epithelium (Barrett’s oesophagus). This represents a risk factor, predisposing to adenocarcinoma transformation

Under normal conditionsAlcolea, M.P., all ingested substances are transported into the stomach in one direction only. The sphincter at the junction between the oesophagus and stomach relaxes to allow food down, remaining closed otherwise to protect the oesophagus from the strong acid composition of the Oesophageal stem cell Stomach digestive secretions. Under certain conditions some of the acid is leaked back into the oesophagus, something known as Gastric reflux (Fig. 10.2) (di Pietro and Fitzgerald 2013). Frequent exposure to this refluxate can lead to oesophageal inflammation, and develop into more advanced oesophageal diseases such as Barrett’s oesophagus (BE), which has the potential to evolve towards oesophageal adenocarcinoma (EAC) as discussed below (Desai et al. 2012).

The continuous harsh environmental conditions to which this organ is exposed dictates the way it is maintained and functionsAlcolea, M.P., and makes it susceptible to abuses exceeding its tolerance that may lead to disease and cancer.

## Oesophageal Cancer

Oesophageal cancer (OEC) represents the eight most common cancer and the sixth most common cause of cancer death worldwide (Rustgi and El-Serag 2014). Despite recent medical advances, this disease still presents late in the clinic and its prognosis remains poor, with a 5 year survival rate of only 10–25% of those diagnosed. There are two major histological subtypes of Oesophageal stem cell, squamous cell carcinoma (SCC) and adenocarcinoma (AC) (Pennathur et al. 2013; Napier et al. 2014).

The incidence of the two major OEC subtypes presents clear geographic patterns that has been attributed to different environmental and nutritional factors. SCC is the major cause of OEC worldwide, representing 90% of all OEC. Oesophageal cancer (OEC) are predominantly high in the so-called Asian belt, encompassing Turkey, northeaster Iran, Kazakhstan, as well as northern and central China. The main risk factors for this type of cancer are tobacco and alcohol consumption; however, other factors such as diet, environmental pollutants and particularly hot beverages have been suggested to influence the distinctive geographic incidence shown by this cancer (Pennathur et al. 2013; Agrawal et al. 2012).

Oesophageal cancer (OEC) has a significantly different etiology to that of SCC. AC has been suggested to arise from abnormal glandular differentiation as a result of long-term gastric reflux (Leedham et al. 2008; Chang et al. 2007). This cancer presents one of the fastest increasing incidences in Europe and Noth America as a result of the rise in obesity, mal-dietary habits and Barrett’s oesophagusBarrett’s oesophagus (BE), a premalignant condition resulting fromAlcolea, M.P. gastric reflux (Pennathur et al. 2013; di Pietro et al. 2014).

## Oesophageal Squamous Cell Carcinoma

SCCs have been associated with a high frequency of Oesophageal squamous cell carcinoma. Recent studies have shown that SCCs in the oesophagus present a greater mutational burden than breast cancer and glioblastoma multiforme (Song et al. 2014). However, the somatic mutation rate was still lower than that observed in head and neck squamous cell carcinomas (Stransky et al. 2011) and oesophageal adenocarcinoma (Oesophageal squamous cell carcinoma) (Dulak et al. 2013).

Different studies in different geographical locations, including North America and China, have identified recurrent genes frequently found mutated in SCC samples. Among those, TP53, NOTCH, PIK3CA and FAT1 (FAT Atypical Cadherin), as well as copy number variations in CCND1 (Cyclin D1) and CDKN2A, seem to be common in the list of SCC mutant genes (Gao et al. 2014; Lin et al. 2014; Zhang et al. 2015; Sasaki et al. 2016).

## Oesophageal Adenocarcinoma

The strongest and best-characterized risk of EAC is gastroesophageal reflux. Decades of evidence have linked EAC to Barrett’s oesophagus (BE), a premalignant condition where the stratified oesophageal epithelium is replaced by a columnar intestinal epithelium in a metaplastic process in response to the strong environmental conditions of chronic gastric reflux. However, despite this knowledgeAlcolea, M.P., Adenocarcinoma has remained cause of concern due to its concerning rise in incidence for the last couple of decades in western and developed Countries. Efforts made to increase detection and surveillance of Barrett’s oesophagus have not significantly affected this trend, given that 95% of EAC arise from patients who had not been previously diagnosed with BE (Reid et al. 2010).

Other Oesophageal adenocarcinoma that increase the risk of EAC are obesity, cigarette smoking and diet low in fruit and vegetables (Engel et al. 2003).

Sequencing studies have described the mutational signature of EAC, reflecting the high mutational burden of this disease. TP53 is the most recurrently mutated gene, other genes mutated at a lower rate inlcude CDKN2A, SMAD4, ARID1A, PIK3CA and SYNE1 (Dulak et al. 2013; Chong et al. 2013). More recently, work from Prof. Fiztgerald laboratory, has shown the highly dynamic nature of the mutational landscape of BE and EAC. This study demonstrated the polyclonal evolution of BE, with high grade dysplasia being able to arise from multiple different clones. This has significant Oesophageal adenocarcinoma, as dysplasia may redevelop from residual BE left behind after treatment therapies (Ross-Innes et al. 2015).

## Understanding Human Oesophagus From Mouse Models

In order to improve the poor prognosis and progressive rise in the incidence of oesophageal cancer, it is imperative to understand the etiology of this complex and heterogeneous disease. Insights as to how it originates and evolves will provide valuable information to unveil new avenuesAlcolea, M.P. for diagnosis and therapeutics.

However, in order to do this, it is first critical to understand how this tissue is maintained under normal homeostatic conditions, how it responds to tissue perturbations such as injury or aggression, and how those rules become deregulated during oncogenesis.

Over last couple of decades there have been several studies trying to unveil the identity of a stem cell population in the oesophagus. Although, there has been some work in human tissue, the most detailed studies use mouse models (Alcolea and Jones 2014). One of the major Human oesophagus is that mice can be manipulated genetically with relative ease (van der Weyden et al. 2002). An increasing range of mouse strains covering a broad spectrum of genetic models have been instrumental in revealing changes in cell behaviour in response to oncogenes, tumour suppressor genes or just simply by allowing visualization of individual cells using fluorescent reporters. These valuable research tools are also extremelyAlcolea, M.P. versatile, making possible the tight control over gene expression in vivo in a temporal, spatial or tissue specific manner, something that has revolutionized our knowledge in epithelial stem cell biology in the last couple of decades (Alcolea and Jones 2013).

Additionally, most of the basic principles of stem cell biology and tumour development have been established in mouse models and have been shown to be conserved from human (van der Weyden et al. 2002; Yuspa et al. 1994). Making the use of mouse models, not only critical tools for basic research, but also for preclinical trials to target specific molecular pathways in order to tests their therapeutic potential.

Given the advantages of available Human oesophagus, it is not surprising that researchers have made use of them to understand oesophageal stem cell dynamics. However, these rodent models also presents some caveats, as fundamental differences between mouse and human oesophagus exist.

**Fig. 10.3:**
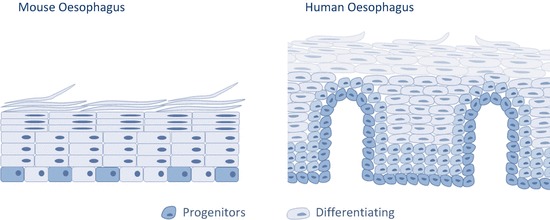
Schematic representation depicting differences between Human and mouse oesophagus

The human oesophagus is a squamous non-keratinized epithelium organized around structures called papillae that divide the tissue into papillary and interpapillary zones (Fig. 10.3). Proliferation takes place in the first 5–6 layers from the basement membrane. On commitment to differentiation, cells exit cell cycle and stratify into the Human oesophagus, migrating to the tissue surface from which they are eventually shed. Unlike the mouse oesophagus, the human oesophagus lacks a cornified protective layer at the tissue surface, making it more vulnerable to the chemical and physical properties of the substances we ingest. This is circumvented to some extent by having additional cell layers that form a thicker epithelium, as well as by the presence of submucosal glands that release mucous and acid neutralizing agents exertingAlcolea, M.P. a protective role (Goetsch 1910; Barbera et al. 2015; Seery 2002; Marques-Pereira and Leblond 1965).

Generally speaking, mouse oesophagus presents a simpler structure. It is also lined by a squamous epithelium that consists of layers of keratinocytes. However, unlike humans, proliferation is confined to the basal cell layer, and no glands, papillae or other accessory structures are found (Doupe et al. 2012; Messier and Leblond 1960; Rosekrans et al. 2015).

Despite the benefit and advantages of using mouse models to understand human oesophageal biology, the significant differences between the two species makes it critical to ultimately test animal observations for their validity in human models.

The recent development of organoid cultures in different epithelial tissues, including the oesophagus, has provided an extraordinary opportunity to translate observations from mouse models into humans (Fatehullah et al. 2016; DeWard et al. 2014; Sato et al. 2011). This 3D in vitro culture method allows for the formation of organized cellular structures with different cellular subtypes and a function reminiscent of that found in the original tissue. This opens new venues to thoroughly characterise human epithelial stem cell biology in health and disease by considering cell-cell interactions while retaining spatial resolutionAlcolea, M.P., at least to some extent.

## Oesophageal Stem Cells in Rodent Models

The oesophagus represent an epithelial barrierOesophageal stem cell in contact with the exterior and, as such, it requires to be in constant turnover to sustain tissue integrity in response to the continuous damage. Proliferation, confined to the basal layer in mice and first few layers in human, is required to generate new cells to maintain the tissue in homeostasis. Under normal conditions, it is critical that upon division the same number of proliferating and differentiating cells are produced in order to maintain a balanced equilibrium. An imbalance will result in the loss of cell production, compromising tissue integrity, or in an excessive cell proliferation potentially leading to cancer (Doupe et al. 2012; Frede et al. 2014; Frede et al. 2016; Alcolea et al. 2014).

Work in the late sixties, studying tritiated thymidine incorporation in the rat oesophagus had suggested that all proliferating cells were equipotent, and that the commitment and exit from the basal layer was stochastic. By performing these experiments, LeblondLeblond, C.P. and co-workers observed how all the cells incorporating the labelled thymidine isotope during division were localized to the basal layer, arguing against asymmetrical division. Over time, half of the labelled cells stratified to the suprabasal layers, suggesting that cell fate making was happening after cell division in a stochastic manner (Marques-Pereira and Leblond 1965).

With the advent of the stem cell/ transit amplifying model proposed to explain epithelial tissue maintenance (Potten and Booth 2002), more recent studies attempted to unveil the identity of a discrete stem cell population in the oesophagus. These hypothesised that the oesophageal epithelium is maintained by a slow-cycling self-renewing stem cell population, generating short lived transit-amplifying cells, that terminally differentiate after a few rounds of division (Croagh et al. 2007). Based on previous studies reporting alpha 6 integrin and CD71 maker combination as a mean to identify epidermal stem cells (Li et al. 1998; Tani et al. 2000), in vivo studies looked into these in mouse oesophagus and concluded that alpha 6 integrin positive basal cells could be separated in two distinct populationsAlcolea, M.P. CD71 dim and CD71 bight. Label retaining and in vivo reconstitution assays indicated that the CD71 dim population fulfilled the criteria of a stem cell compartment (Croagh et al. 2007). However, this population failed to manifest an enhanced colony forming potential in in vitro clonogenic assays.

A subsequent study used a Hoechst exclusion assay to identify a label retaining population in the mouse oesophagus that was enriched for CD34 expression; a known stem cell marker (Trempus et al. 2003). This population presented increased clonogenic and regenerative potential both in vitro and in vivo, showing the typical features of a potential stem cell population. Interestingly, further analysis of this putative stem cell population did not correlate with the integrin alpha 6 high/CD71 dim expression profile previously reported in mouse oesophageal stem cells (Kalabis et al. 2008).

A more recent report from DeWardDeWard, A.D. et al. used a combination of basal cell surface markers to separated oesophageal cells into distinct populations with different in vitro organoid forming efficiency. This study shows that SOX2 is oesophageal basal cell maker that plays an important role in organoid formation and self-renewal. And suggests basal cells expressing the highest levels of basal markers integrin alpha 6, beta 1 and p75 represent a putative stem cell population based on their increased organoid formation efficiency. However, no differences were observed in their self-renewal potential (DeWard et al. 2014). Based on this observation, the study concludes that a non-quiescent stem cell population resides in the basal epithelium of the mouse oesophagus.

The development of new genetically engineered mouse strains expressing multicolour fluorescent reporters which expression may be controlled temporally and/or spatially by specific promoters and/or drug treatment, has revolutionized our knowledge of cell behaviour in epithelial tissuesAlcolea, M.P. in health and disease (Alcolea and Jones 2013). By exploiting the available reporter mouse strains scientists can now label individual cells throughout the tissue with an inheritable fluorescent reporter, and track their fate over the course of time either by performing end point experiments, or by in situ live imaging in the living organism (Alcolea and Jones 2014; Park et al. 2016).

Using Quantitative methods of Lineage tracing, we performed a comprehensive study to reconcile previous observations on mouse oesophageal stem cell behaviour. Individual basal cells were fluorescently labelled, and their fate tracked over the course of 1 year. Large scale clone size analysis using methods of mathematical statistics revealed that a single progenitor population that divides stochastically, balancing the production of proliferating and differentiating cells, is responsible for the maintenance of the mouse oesophageal epithelium (Doupe et al. 2012). Additional, transgenic label-retaining assays based on calculating the dilution of doxycycline induced Histone-2B-GFP fusion protein (Tumbar et al. 2004) indicated that no slow-cycling epithelial cells were present in the oesophageal epithelium. Further, quantification of the Histone-2B-GFP levels in individual cells led to the conclusion that all basal cells divide at a similar rate, in agreement with the original observations by LeblondLeblond, C.P. and co-workers (Marques-Pereira and Leblond 1965; Doupe et al. 2012).

**Fig. 10.4:**
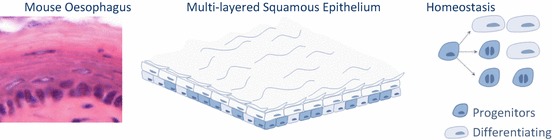
Stochastic model of oesophageal tissue maintenance in mouse. Quantitative cell fate analysis in the mouse oesophagus has revealed that a single functionally equivalent progenitor population maintains the tissue by dividing stochastically, balancing the production of proliferating and differentiating cells. Each division can produce one of three outcomes. Symmetric fate results in two proliferating or differentiating cells, while asymmetric divisions generate one of each

In order to unveilAlcolea, M.P. whether tissue injury could reveal populations with distinctive regenerative potential, a refined endoscopic method was used to create a discrete incision in the mouse oesophagus. Similar genetic Lineage tracing and label retaining assays were performed. Remarkably, the uniformity of the basal cell population was once more revealed in response to wounding. The widespread activation of progenitor cells around the wound rapidly produced an excess of proliferating cells in order to close the defect in the epithelium (Fig. 10.4), leading to a very efficient and rapid healing response (Doupe et al. 2012).

## Oesophageal Stem Cells in Human

As discussed above mouse and human oesophagus show certain histological differences. In essence, both tissues are formed by layers of squamous epithelial cells divided in two main compartments; the basal zone with one (mouse) or several (human) layers of small basophilic cells, and the differentiated zone where cells become progressively flatter as they approach the lumen surface where they shed from the tissue. One of the major differences in human oesophagus is the existence of a structured architecture around papillae. These arise from the invagination of the lamina propia at regular intervals and results in the tissue being divided in papillary and interpapillary epithelium (Fig. 10.3) (Goetsch 1910; Seery 2002). These defined structures have been proposed to be a potentialAlcolea, M.P. niche for stem cells in the human oesophagus (Barbera et al. 2015; Seery 2002; Seery and Watt 2000). However, such compartmentalization is not found in mice (Doupe et al. 2012).

The number of studies available on human oesophagus have been limited by the inaccessibility of the sample, as well as the technical challenges to study stem cell behaviour and regeneration in this tissue.

Initial studies, based on PCNA staining, a proliferation maker, suggested the existence of a putative stem cell population located at the tip of the papillae (Jankowski et al. 1992). Later studies looked into cell division symmetry and found that cells in the interpapillary zone divided rarely and asymmetrically; giving rise to one basal daughter and one suprabasal differentiating cell (Seery and Watt 2000). They concluded that interpapillary basal cells attained to the expected stem cell definition at the time; stem cell fate in squamous tissue was believed to be maintained largely through division asymmetry (Watt and Hogan 2000).

More recently label retaining assays using the thymidine analogue 5-iodo-2′deoxyuridine (IdU) in patients undergoing oesophagectomy showed a higher proportion of IdU retaining cells in the papillary basal layer of healthy oesophagus. The conclusion was that a putative slow-cycling self-renewing stem cell population resides in the defined niche of the oesophageal papillae (Pan et al. 2013).

The most recent report studying human oesophageal tissue maintenance, uses a comprehensive Wholemount staining technique to asses for proliferation and stem cell markers such as CD34. Data shows that proliferation and mitotic activity was highest in the interpapillary basal layer and decreased linearly towards the tip of the papilla, where a CD34 positive population resides. Additional 2D and 3D organotypic in vitro assays looked into the regenerative potential of differentAlcolea, M.P. cell populations sorted based on CD34 and epithelial cadherin. Interestingly, no differences in self-renewal were observed when performing either single cell or population assays (Barbera et al. 2015). These observations are in agreement with earlier studies suggesting a slow cycling population resides in the papillary zone, and seem to resolve conflictive reports (Pan et al. 2013; Jankowski et al. 1992). Interestingly, this study also presents data in line with recent findings in mouse oesophagus. Progenitor cells, which can respond to injury and regenerate tissue, were found to be widespread and are not restricted to the basal layer, including cells that have already committed to epithelial differentiation (Barbera et al. 2015; Doupe et al. 2012).

## Oesophageal Cell Behaviour in Tumourigenesis

The advent of in vivoAlcolea, M.P. linage tracing techniques has represented a powerful technique to start understanding changes in oesophageal cell behaviour in response to mutations and mutagens that favour tumour development.

**Fig. 10.5:**
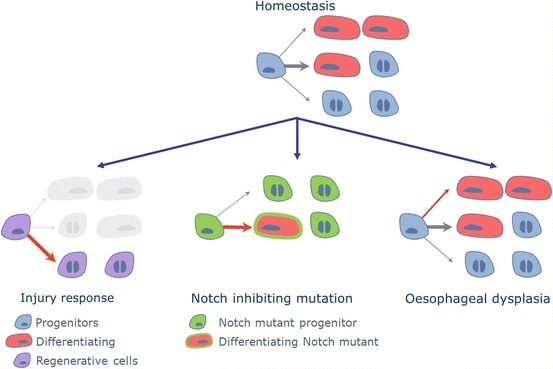
Oesophageal progenitor cells are able to change their cell fate programme in response to tissue perturbations such as injury, neoplastic mutations and tumorigenesis (*red arrows*). Notch inhibiting mutations in progenitors showed an increased proliferation rate, favouring asymmetric cell division. Surprisingly, benign tumours developed upon cigarette smoke derived nitrosamines did not show a significant change in the rate of cell division. The perturbation seemed to be the result of a discrete bias towards proliferation

Sequencing studies previously suggested that loss of function Notch mutations and loss of heterozygosity were frequently found in squamous cell carcinomas, including oesophageal SCCs (Agrawal et al. 2011, 2012; Song et al. 2014; Stransky et al. 2011; Gao et al. 2014; Lin et al. 2014). Using a Lineage tracing approach similar to that previously used to study mouse oesophageal tissue maintenance (Doupe et al. 2012), we challenged mouse oesophageal homeostasis by inhibiting Notch signalling in vivo. An engineered mouse model expressing an inducible dominant negative form of mastermind like-1 tagged to a fluorescent GFP reporter (DNM1-GFP) was used in this study (Tu et al. 2005). Quantitative clonal data revealed that Notch inhibition confers a strong competitive advantage to mutant progenitor cells, generating clones that expand rapidly over the weeks following induction. Further analysis on clonal growth and progenitor differentiation suggested that mutants present a blockage in terminal division, where dividing cells produce two differentiating cells (Fig. 10.4). As a result, mutant cells divide 3 fold faster than wild type cells, and, on average, each cell division produces an excess of progenitors over differentiating cells (Fig. 10.5) (Alcolea et al. 2014). Interestingly, the clonal advantage of these clones does not only rely on cell autonomous mechanisms but also exerts a ‘Bystander effect’, actively eliminating wild type cells, similar to those observed in super competitor mutants in Drosophila (de la Cova et al. 2004; Moreno and Basler 2004). Additional treatment with carcinogens illustrates the potential role of Oesophageal cell behaviour in tumour formation; mutant clones were seen to provideAlcolea, M.P. means for other less advantageous mutations to colonize the tissue when co-existing (Alcolea et al. 2014). This exemplifies how different mutations could potentially synergise during the early stages of tumour formation.

Further studies have used a combination of Diethylnitrosamine (DEN) and the multikinase inhibitor Sorafenib, as a two-stage carcinogenic protocol to investigate cell dynamics during oesophageal tumorigenesis. Oesophageal cell behaviour is a nitrosamine found in cigarette smoke and traditionally used to induce tumours in the oesophageal epithelium (Hoffmann et al. 1982; Rubio 1983; Rubio et al. 1987). These Nicotine-derived compounds are activated in the body to form alkylating agents that cause DNA damage (Goodsell 2004). Inclusion of Sorafenib was drawn from previous observations showing the cancer promoting effect of this drug. Sorafenib was shown to lead to SCC formation in skin, and head and neck in patients treated for liver, kidney and thyroid cancers (Schneider et al. 2016; Arnault et al. 2009). DEN and Sorafenib drug combination generates early tumours forming high grade dysplasias (HGD) in the mouse oesophagus. Interestingly, lineage tracing data in the epithelial compartment points to the polyclonal origin of these tumoral lesions. Against all predictions, cells in dysplasias shared a common dynamics, with progenitor cells showing a moderate bias towards the production of dividing over non-dividing daughter cells (Fig. 10.5). Also, despite the tumour outgrowth no significantAlcolea, M.P. change in the rate of cell division was observed (Frede et al. 2016).

The remarkably uniform behaviour described in Oesophageal cell behaviour contrasts with observations in squamous cell carcinomas produced in a Kras G12D mutant background. These advanced cancers were characterised by the existence of a subset of clones with a significant bias towards proliferation, reflecting the onset of cancer heterogeneity. It remains to be elucidated whether this subpopulation has an increased tumour initiating potential when compared to the bulk tumour cell population (Frede et al. 2016).

## Oesophageal Cancer and Microenvironment

Cancer is a complex disease that develops in response to a concert of genetic alterations and environmental factors. The constant exposure of the oesophagus to damaging agents through ingestion, as well as gastric refluxate may result in tissue injury. This can have a significant impact on the epithelium not only by promoting the mutational burden, but also indirectly, by activating the underlying stroma (Lin et al. 2016).

It has traditionally been thought that the sole cause of cancer lays on the accumulation of genetic alterations that promote disease progression. However, increasing evidence suggest that there is an entire new dimension to it, i.e. the tumour microenvironment. Non-cell autonomous components, coming from the stroma, can significantly contribute not only to cancer progressionAlcolea, M.P. but also to cancer initiation (Hu et al. 2012; Whiteside 2008; Tlsty and Coussens 2006).

The primary function of the stroma is to offer structural support to organs and epithelial tissues lining them. However, it also serves as a sensor orchestrating the signals required to modulate cell behaviour in response to environmental changes. Communication between epithelial and stromal cells is essential for tissue damage repair. However, stromal activation can be aberrantly triggered by the abnormal behaviour of mutant epithelial cells, misleadingly understood as an injury, promoting tumorigenesis (Arwert et al. 2012).

The tumour stroma, which consists of immune cells, fibroblasts, endothelial cells, perivascular cells, adipocytes and extracellular matrix, constitute the microenvieronment in which the tumour must develop (Arwert et al. 2012). Given that tumours have been proposed to function as an injury that is not able to heal, suggested by DvorakDvorak, H.F. (Dvorak 1986), the interplay between tumour cells and the different stromal compartments will have a significant role in tumour development and progression. The same way this interplay is central for adequate wound repair. The main difference resides in the fact that wound healing is a controlled mechanism, while tumour formation is a disorganized process (Arwert et al. 2012; Gurtner et al. 2008).

Among the risk factors promoting oesophageal cancer discussed above, cigarette smoke, alcohol, gastric reflux, obesity and dietary habits, all of them share a common feature. They all have a significant impact on the tumour stroma, mainly by promoting tissue damage. This has the inevitable consequence of fibroblast activation, increased immune response, changes in extracellularAlcolea, M.P. matrix and vascular reorganization, among others (Lin et al. 2016).

### Mesenchymal Compartment

The main cellular component of the tumour stroma in most tumour types are fibroblasts. Tumour associated fibroblasts (TAFs) have been shown to be a heterogeneous cell population that plays an active role from the earliest stages of tumour formation. TAFs contribute to disease progression by providing the suitable environment for carcinogenesis, proliferation, angiogenesis and invasion. Growth factors, cytokines and extracellular matrix are released to promote tumour cell transformation (Joyce and Fearon 2015; Malanchi et al. 2012; Zhang and Wang 2015; Kalluri 2016). More recently, it has been shown how TAFs can also have an impact on drug resistance by signalling to tumour cells (Hirata et al. 2015; Au Yeung et al. 2016; Kaur et al. 2016).

Cancer associated fibroblasts (CAFs) have been proposed to have a critical role in the development of oesophageal cancer. Reports suggests that oesophageal CAFs can derive from different cellular populationsAlcolea, M.P., including normal fibroblasts and bone marrow-derived cells among others (Nouraee et al. 2013; Hutchinson et al. 2011). Transforming growth factor β1 (TGFβ1) and microRNAs have been implicated in the conversion of fibroblasts to CAFs (Noma et al. 2008; Tanaka et al. 2015).

In the oesophagus, fibroblasts are localized in the submucosa layer laying directly underneath the epithelial mucosa (Goetsch 1910). Increased transforming growth factor β1 (TGFβ1) and hepatocyte growth factor (HGF) have been linked to the progression from dysplasia to ESSC (Xu et al. 2013). In human ESCC, TGFβ receptor II (TβRII) was found to be downregulated in CAFs. This was associated with increased proliferation and reduced apoptosis in adjacent epithelial cells (Achyut et al. 2013). Incresed cyclooxygenase (COX)-2, the enzyme of prostaglandin E2 (PGE2), has been linked to both ESCC and EAC development via its pro-inflammatory function (Achyut et al. 2013; Taddei et al. 2014). Indeed, one of the means by which CAFs have been proposed to contribute to carcinogenesis is by producing pro-inflammatory factors.

### Immune Compartment

One microenvironmentalAlcolea, M.P. component that has become increasingly relevant in recent years due to mounting evidence probing its significant contribution to tumorigenesis and its therapeutic potential is the immune compartment (Chen and Mellman 2017).

Injury by Gastric refluxate in the oesophagus has been shown to result in chronic inflammation with upregulation of cytokines, such as IL1b, IL6, and IL8 (Fitzgerald et al. 2002). Increased IL1b/IL6 signalling contributes to the metaplastic and dysplastic conversion of BE, as well as its evolution towards oesophageal adenocarcinoma (Quante et al. 2012). A mouse model overexpressing interleukin-1b developed human Barrett-like metaplasia and EAC in an interleukin 6 dependent manner. This phenotype was accelerated by exposure to bile acids, one component of gastroduodenal reflux, or nitrosamines, generated at the oesophageal junction when salivary nitrite is reduced in response to gastric secretions (Winter et al. 2007). Lineage tracing data suggested that Lgr5 positive cells of gastric origin were the origin of the Barrett’s lesion in this IL1b-IL6 immune permissive environment. The results also indicated that the oesophageal to columnar transition happens under the control of Notch Delta1-dependent signalling (Quante et al. 2012).

The role of inflammation is also important for ESCC (Sadanaga et al. 1994). It has been shown that the main risk factors for this type of cancer, smoking and alcohol, favour an inflammatory response via direct chemical irritation of the oesophageal epithelium, as well as production of reactive oxygen species (Sadanaga et al. 1994; Kubo et al. 2014). A conditional mouse model where p120catenin was lost in the oesophagus revealed the role of the tumour microenvironment as a tumour driver. ESSC development in this modelAlcolea, M.P. was associated to inflammation, immune cell infiltration, and increased NFkB/Stat-3 cross-talk in tumours (Stairs et al. 2011). A later study reinforced the important role of the immune response in ESCC development. Conditional SOX2 overexpression in the oesophagus was insufficient to drive SCC formation. Transformation of oesophageal progenitor cells required cooperation of increased Sox2 and microenvironment-activated Stat3, leading to tumorigenesis (Liu et al. 2013).

Several immune cell types have been involved in tumour development. Although the main function of our immune system is to protect our organism from invasion, the same must have mechanisms that protect us against persistent or dysregulated immune reactions. This is a critical function for our survival. Tumour cells have been proposed to hijack some of these mechanisms in order to persist and evolve. For instance, regulatory T cells that under normal conditions maintain tolerance to self-antigens, preventing autoimmune disease, if aberrantly activated in response to oesophageal cancer, promote tumour immune suppression favouring disease progression (Nabeki et al. 2015). Myeloid-derived suppressor cells (MDSCs) are immature cells that also suppress the immune reaction by induction of regulatory T cells, and inhibition of protective cell types such as T cells and natural killer cells. This cell population was found to be increased in ESCC mouse models (Stairs et al. 2011; Chen et al. 2014). Similarly, macrophages switching from M1 to M2 start producing cytokines and growth factors that favour oesophageal tumour development (Miyashita et al. 2014). Another immune suppressive mechanism hijacked by cancer cells is the modulation of immune check-points. Programmed cell death protein ligand (PD-L1) is a protein expressed on the surface of several tumour cells, and it is thought to play a role in immune escape by inhibiting T cell function. PD-L1 has shown a significant potential as melanoma target treatment and also presents good prospects for oesophageal cancer (Raufi and Klempner 2015).

Ultimately, cancer represents a complex interplay between malignant cells and their neighbouring stromal compartment. The realisation of the increased genetic stability of stromal cells compared to cancer cells has made them an attractive cellular compartment, formerly disregardedAlcolea, M.P.. Mounting line of evidence indicate the largely unexplored potential of tumour microenvironment, not only as a source of plausible therapeutic targets, but also of diagnostic and prognostic markers (Lin et al. 2016).

All in all, the oesophagus has proven to be an excellent model to understand basic epithelial stem cell biology. Its multi-layered stratified architecture, constant turnover, interaction with the environment and cross-talk with the microenvironment render this an ideal tissue where to explore stem cell behaviour in health and disease. Despite good progress in the field, further research is still needed to identify how stromal changes govern epithelial cell behaviour, and how those contribute to cancer development. The new research tools now widely accessible, such as a broad spectrum of genetically engineered mouse models, organoid cultures and recent developments in CRIPR technology represent an exciting prospect for oesophageal stem biology.
